# Genomic Characterization of a Prophage, Smhb1, That Infects *Salinivibrio kushneri* BNH Isolated from a Namib Desert Saline Spring

**DOI:** 10.3390/microorganisms9102043

**Published:** 2021-09-28

**Authors:** Israel Olonade, Leonardo Joaquim van Zyl, Marla Trindade

**Affiliations:** Institute for Microbial Biotechnology and Metagenomics (IMBM), University of the Western Cape, Bellville, Cape Town 7535, South Africa; israelolonade@outlook.com (I.O.); ituffin@uwc.ac.za (M.T.)

**Keywords:** *Salinivibrio*, myoviridae, Namib, playa, phage

## Abstract

Recent years have seen the classification and reclassification of many viruses related to the model enterobacterial phage P2. Here, we report the identification of a prophage (Smhb1) that infects *Salinivibrio kushneri* BNH isolated from a Namib Desert salt pan (playa). Analysis of the genome revealed that it showed the greatest similarity to P2-like phages that infect *Vibrio* species and showed no relation to any of the previously described *Salinivibrio*-infecting phages. Despite being distantly related to these Vibrio infecting phages and sharing the same modular gene arrangement as seen in most P2-like viruses, the nucleotide identity to its closest relatives suggest that, for now, Smhb1 is the lone member of the *Peduovirus* genus *Playavirus*. Although host range testing was not extensive and no secondary host could be identified for Smhb1, genomic evidence suggests that the phage is capable of infecting other *Salinivibrio* species, including *Salinivibrio proteolyticus* DV isolated from the same playa. Taken together, the analysis presented here demonstrates how adaptable the P2 phage model can be.

## 1. Introduction

With an estimate of ~10^31^ viral particles present in the environment, numbering from 10^7^ to 10^9^ per gram of soil or sediment [1,2] to 10^10^ per liter of sea water [3], bacteriophages (viruses that infect bacteria) are known to be widely distributed in nature. While the discovery of phages and the investigation of phage model systems have contributed to our understanding of molecular biology, only more recently has their impact on ecosystems been fully appreciated [4,5]. 

The range of interactions phages can have with their hosts suggests that they can play an important role in shaping microbial diversity and ecology [6,7,8]. Such roles include significant contributions to nutrient cycling in the ocean by being major predators of bacteria and archaea [9] and as major contributors to genetic exchange in the environment with phages estimated to transduce 10^25^ to 10^28^ base pairs of DNA per year in the marine environment alone [10].

Haloalkaline environments are considered to have the highest abundance and diversity of viruses of all types of environments, with the highest viral count documented to be 2 × 10^9^ per mL in the hypersaline Mono Lake, California [11]. The impact phages can have on trophic levels was demonstrated in the East African Rift Valley lakes where a short food chain, involving cyanophages, the cyanobacterium *Arthrospira fusiformis*, and the Lesser Flamingo, exists [12]. 

The population of *A. fusiformis* can collapse drastically and unpredictably due to a cyanophage infection, and this fluctuation represents the major factor influencing the population and distribution of the Lesser Flamingo [13,14]. The study also made an interesting observation that, although the phage to bacteria ratio constantly fluctuated, the difference remained in the same order of magnitude. This suggests that phages are actively involved in keeping bacterial populations at a threshold.

The Namib Desert salt playas are unique environments with salinities in the range of 3–15% depending on the sampling depth, evaporation rate as a consequence of the time of the day, and the distance from the source [15,16,17,18]. The Eisfeld site explored here has a salinity range of 4.5–8.6% [19]. These values are relatively low in comparison to other saline environments, such as the moderately hypersaline Salton Sea (CA, USA), which has a salinity range of 5% (water table) to 11.8% (sediments) [20] or crystallizer ponds and solar salterns with a range from 13.8% to 37% [21,22]. 

Physico-chemical analysis also showed that the site has a pH range of 6.5 (source) to 8.5 (sink), a conductivity range of 66–180 mS/cm, and total dissolved solids (TDS) range of 42,000–115,000 ppm [15,18,19]. Temperatures have also been reported to reach up to 50 °C [15]. These parameters together with its unique, under sampled, geographic location suggests that it could be an environment that harbors novel halotolerant and halophilic bacteria, which could host potentially unique viruses.

A description of the virome data for the Eisfeld site was presented elsewhere [19] with the families *Circoviridae*, *Microviridae*, and *Inoviridae* found to comprise a significant portion of the viral diversity of the site. The genus *Salinivibrio* is comprised of halophilic, curved rod shaped, motile, facultative anaerobic, Gram-negative bacteria that, according to the genome taxonomy database classification, currently consists of seven species. Phages CW02, G3, and UTAK are the only phages known to infect the *Salinivibrio* species; however, only phage CW02 has been described in terms of general features and genomic architecture, while the host for UTAK may have been incorrectly reported [23,24]. 

Recent efforts by the bacterial and archaeal virus subcommittee of the ICTV has seen a large number of phages being classified and re-classified, and the P2-like viruses are no exception. Within the last four years, the subfamily has expanded from two genera/nineteen species in 2017 to thirty-one genera/seventy-six species in 2020 and a proposal in 2021 to elevate the subfamily to family status [25]. Here, we describe the isolation of a new P2-like phage, Smhb1, which infects a *Salinivibrio* species that, given the seeming rarity of *Salinivibrio*-infecting phages and its novel nucleotide sequence compared against newly classified P2-like viruses, makes it highly unique.

## 2. Materials and Methods

### 2.1. Sampling and Bacterial Isolation and Identification

Samples were collected from the Eisfield salt playa 21.5 km Northeast of Swakopmund (22°29’5.31” S; 14°34’17.88” E), Namibia. Fifty milliliters of the microbial mat suspended in spring water from the playa was shaken at 50 rpm for 2 h to encourage the dissociation of bacterial cells from biofilm debris. After a low-speed centrifugation (26.5× *g* for 10 min), 10 µL of the supernatant was spread on MHB agar plates containing the following per liter: 98 g NaCl (Cat. No. S9888), 20 g bacteriological agar, 10 g yeast extract, 5 g tryptone, 2 g KCl (Cat. No. P3911), 1 g D-glucose (Cat. No. SAAR2676020EM, Merck, Darmstadt, Germany), 1 g MgSO_4_·7H_2_O (Cat. No. M2773), 0.36 g CaCl_2_·6H_2_O (Cat. No. 442909), 0.23 g NaBr (Cat. No. 310506,), 0.06 g NaHCO_3_ (Cat. No. S6014), and 0.001 g FeCl_3_ (Cat. No. 157740). 

Except for the yeast extract (Cat. No. HG000BX6.500), bacteriological agar (Cat. No. HG000BX1.500), and tryptone (Cat. No. HG000BX4.250) powder purchased from biolab (Merck, Modderfontein, South Africa), the other reagents were purchased from Sigma-Aldrich, St. Louis, MO, USA. The plates were incubated at 37 °C until colonies were observed. Colonies of varying morphologies were selected after visual inspection and Gram staining. Each isolate was purified by three rounds of re-streaking on fresh agar plates and stored in MHB broth supplemented with 50% glycerol at −80 °C until required.

The 16S rRNA gene from each isolate was amplified using the universal primers E9F (5′-GAGTTTGATCCTGGCTCAG-3′) [26] and U1510R (5′-GGTTACCTTGTTACGACTT-3′) [27]. DNA templates were prepared by re-suspending a loop-full of each isolate in 100 μL of PCR grade water and boiling at 85 °C for 5 min. Each PCR reaction contained 12.5 μL of KAPA Taq ReadyMix (Cat. No KK1006, Kapa Biosystems, Boulder, CO, USA), with a 0.5 µM final primer concentration and 2 μL of DNA template that was adjusted to a final volume of 25 μL with water. 

The PCR conditions were an initial denaturation at 95 °C for 3 min, followed by 35 cycles of denaturation at 95 °C for 30 s, annealing at 55 °C for 30 s, and extension at 72 °C for 2 min, with a final extension at 72 °C for 2 min. DNA bands were excised and purified using the Nucleospin^®^ Gel and PCR Clean-up kit (Cat. No. 740609.50, Macherey-Nagel, Dueren, Germany) according to the manufacturer’s instruction. The amplicon sequence was determined using an ABI PRISM^®^ 377 automated DNA sequencer (Applied Biosystems, Thermo Scientific, Waltham, MA, USA) at the Central Analytical Facility of the University of Stellenbosch, South Africa.

### 2.2. Smhb1 Isolation and Transmission Electron Microscopy

During routine culturing of bacterial isolates identified as *Salinivibrio* species, culture lysis was observed for *S. kushneri* BNH, and epifluorescence microscopy confirmed the presence of virus-like particles in the culture supernatant. Plating a lawn of the culture also yielded individual plaques. Phage samples were prepared for imaging by centrifugation of the phage lysate at 27,200× *g* (Centrifuge Eppendorf 5417 R, Merck, Darmstadt, Germany) for 5 h and the phage pellet resuspended in 100 mM ammonium acetate, pH 6.5 and washed four times in this solution, according to [28]. 

Three microliters of the phage suspension were spotted onto glow-discharged (EMS100 Glow Discharge Unit, Electron Microscopy Sciences, Hatfield, PA, USA) 200-mesh carbon-coated copper grids (Cat. No. AGG2200C, Agar Scientific, Stansted, Essex, UK) and allowed to adsorb for 1 min. The grid was washed twice in sterile water and stained with 2% aqueous uranyl acetate (SPI Supplies, West Chester, PA, USA) for 30 s. The samples were viewed using a LEO 912 Omega TEM (Zeiss, Oberkochen, Germany) at 120 kV, and images were collected using a ProScan CCD camera (Cincinnati, OH, USA).

### 2.3. Genomic DNA Extractions

To extract phage genomic DNA, the supernatant of a 48-h 1 L culture of the bacterial host was cleared by centrifugation at 15,971× *g* for 30 min. Debris was further removed by passing it through a 0.22 μm filter (Cat. No. FJ25ANCCA002DD01, GVS, Bologna, Italy). Phage particles were concentrated by adding PEG 8000 (89510-1KG-F, Sigma-Aldrich, St. Louis, MO, USA) to a final concentration of 7% (*wt*/*vol*) and then incubated overnight at 4 °C. Viruses were pelleted at 9585× *g* for 15 min and resuspended in 1 mL SM buffer, and the DNA was extracted using a modified version of a published protocol [29]. Briefly, 1 mL of the resuspended viruses was transferred to a 15 mL Greiner centrifuge tube (T1943, Sigma-Aldrich, St. Louis, MO, USA) and 12.5 µL of 0.1 M MgCl_2_ was added to stabilize the DNA. Five microliters of DNAse I (1 U/uL, (Cat. No. EN0521, Fermentas, Vilnius, Lithuania) and 1 µL of RNAse A (10 mg/mL, (Cat. No. EN0531, Fermentas, Vilnius, Lithuania) were added, and the mixture incubated at 37 °C for one hour to digest the host DNA and RNA. The absence of contaminating DNA was confirmed by PCR amplification of the 16S rRNA gene as described above. 

Proteinase K (final concentration 97.5 µg/mL, Cat. No. EO0491, Fermentas, Vilnius, Lithuania), 40 µL of 0.5 M EDTA and 50 µL of 10% SDS were added, and the mixture was incubated at 55 °C for 2 h with periodic mixing by gentle inversion. Complete lysis of the phage particles was confirmed by adding 1 µL of 10,000 × SYBR Gold (Cat. No. S11494, Invitrogen, Waltham, MA, USA) to 10 µL of the mixture with observation under a fluorescence microscope (Zeiss Axioplan 2, Zeiss, Oberkochen, Germany). 

Viral DNA was extracted by adding an equal volume of Phenol: Chloroform: Isoamyl alcohol (25:24:1) and gently inverting the tubes. Phases were separated by centrifugation (Eppendorf 5810 R) for 5 min at 17,900× *g* at room temperature. The top aqueous layer was carefully removed and transferred to a sterile 2 mL Eppendorf tube. DNA was precipitated overnight at 4 °C by adding two volumes of absolute ethanol and 1/10 volume of 3 M NaOAc (pH 5.2), followed by pelleting at 17,900× *g* for 10 min. The DNA pellet was resuspended in 50 µL of TE buffer (pH 8).

Bacterial genomic DNA was extracted from the two *Salinivibrio* species (DV and BNH) isolated from the saline site. Five milliliters of an overnight culture of each strain in MHB broth was centrifuged at 2700× *g* for 5 min to collect bacterial cells. Genomic DNA was extracted from the bacterial pellets using a modified protocol [29]. Briefly, each pellet was resuspended in 950 µL of TE buffer and 50 µL of 10% SDS, and 5 µL of 20 mg/mL proteinase K (Fermentas) were added. 

The suspension was incubated at 37 °C for 1 h. One hundred and eighty microliters of 5 M NaCl and 150 µL of CTAB/NaCl (10%/4% *wt*/*vol*) solution were added, mixed thoroughly, and incubated at 65 °C for 20 min. An equal volume of chloroform/isoamyl alcohol (24:1) was added. The solution was mixed gently and centrifuged at 20,800× *g* for 10 min at room temperature to separate the phases. 

The DNA-containing supernatant was transferred to a fresh tube and extracted with an equal volume of phenol/chloroform/isoamyl alcohol, mixed gently, and centrifuged at 20,800× *g* for 10 min at room temperature. The clear upper phase was transferred to a new tube, 0.6 volume isopropanol was added, and the sample was gently mixed and centrifuged at 20,800× *g* for 10 min at room temperature. The pellet was washed with 70% ethanol, air dried, and resuspended in 200 µL of 1X TE buffer (pH 8).

### 2.4. Whole Genome Sequencing and Analysis

Prior to sequencing, the bacterial and phage genomic DNA was further cleaned using the Qiagen Gel Extraction kit (Qiaex II, Cat. No. 20021). Sequencing libraries were prepared using the Illumina Nextera XT library prep kit as per the manufacturer’s instructions, and sequencing was performed using the Illumina MiSeq Reagent kit V3, which included a 10% phiX V3 spike (Preparation guide, Part #15031942, May 2012 revision). This resulted in 2 × 300 bp paired end reads (forward and reverse). A de novo assembly was performed on reads using CLC Genomics version 7.5.1 (Qiagen, Venlo, Netherlands) at the default settings. 

For the genomes of DV and BNH, annotation of the assembled contigs were carried out using the NCBI prokaryotic genome annotation pipeline (PGAP) [30]. Tetranucleotide usage deviation was calculated using TETRA in JSpeciesWS [31]. Phylogeny for bacterial genomes was established using GTDB-Tk through its implementation in K-Base [32,33]. Clustered regularly interspaced short palindromic repeats (CRISPRs) regions were scanned for using CRISPRFinder [34]. 

For phage Smhb1, the longest assembled contig was uploaded to the phage search tool PHASTER [35] for early identification of phage genes, followed by BLASTx comparison of non-coding segments against the NCBInr database. These were then manually curated using the CLC Genomics workbench and BLAST. Closely related phages were identified by examining data generated using VIRIDIC [36] and published in ICTV taxonomic proposal 2020.117B.R.*Peduovirus*es. 

Comparison of genomic synteny was performed using Easyfig [37] using the tBLASTx function with the settings: min. length 35; max. e-value 0.001; min. identity value 0. VipTree [38] was used for comparison of Smhb1 proteins to those of other *Peduovirus*es. Direct repeats were identified using REPFIND [39] with a 10-bp minimum repeat length. tRNA genes were predicted using ARAGORN [40] and the tRNAscan-SE program [41,42]. Intron prediction was done using the RNAweasel server [43]. Transmembrane regions were predicted using the TMHMM server v2.0 [44]. 

For phylogenetic tree construction, the full-length amino acid sequences of selected terminase large subunit proteins were aligned using MUSCLE in MEGA7 [45], and the tree was constructed using the built-in program [46]. To determine amino acid usage profiles, the major capsid protein, terminase large and small subunits, sheath protein, head completion scaffold protein, and portal protein sequences were concatenated, and duplicate sequences removed using CD-HIT [47]. 

The sequences were aligned in MEGA 7 using MUSCLE and the alignment was manually curated to remove gaps (p-distance = 0.51). The aligned proteins were split into representatives from moderate halophiles (those infecting *Vibrio* sp., SMHB1 and C5a) and non-halophile infecting phages and these were used as “query” and “background” inputs to Composition Profiler, respectively [48].

## 3. Results and Discussion

### 3.1. Host Isolation and Sequencing; Smhb1 Morphological Characterization, and Host Range Testing

**Figure 1:**
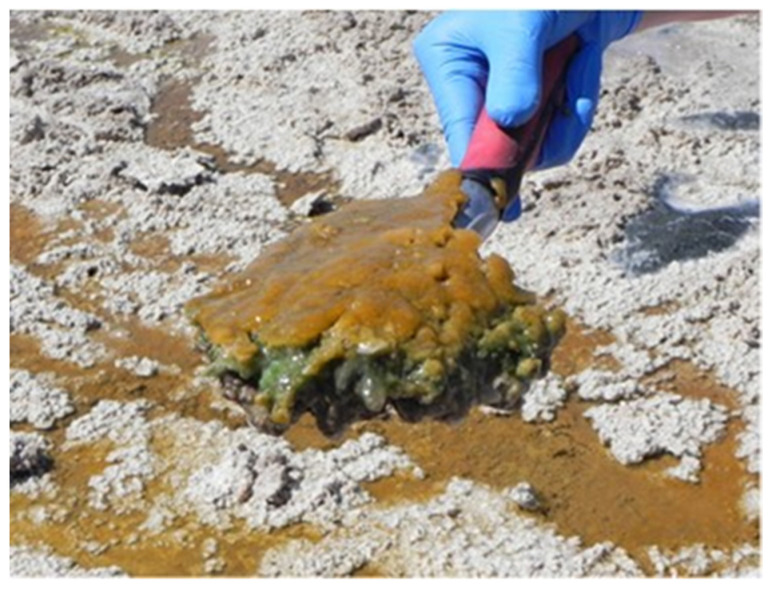
Microbial mat sample from the Eisfeld site.

**Figure 2:**
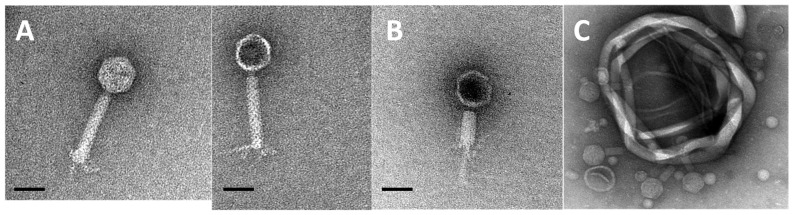
Electron micrographs of phage Smhb1. (**A**) Intact viral particles. (**B**) Phage with contracted tail and that has presumably ejected the genome. (**C**) Several phage particles bound to cell debris. Images in (**A**) and (**B**) are at 50,000× magnification, and the scale bar represents 50 nm.

A number of bacteria (Halomonas caseinilytica, Halomonas eurihalina, Halomonas sinaiensis, Idiomarina loihiensis, Marinobacter xestospongiae, and Virgibacillus salarius) including two Salinivibrio species (designated DV and BNH throughout the manuscript) were isolated from dense microbial mats collected from the Eisfeld playa (Figure 1). Based on the spontaneous development of plaques when plating the BNH strain during routine culturing, we sought to investigate the presence of a prophage present in this isolate. Following lytic induction and washing to prepare the phage for TEM, the morphology was determined as a myovirus with a head-neck-contractile tail geometry [49] (Figure 2). The phage had a capsid diameter of approximately 56 nm, within the range of 30 to 60 nm reported for 65% of aquatic viruses [50], and a tail length of approximately 106 nm.

We assessed the sensitivity of the isolates mentioned above as well as the *Vibrio/Salinivibrio* species listed in Table S3 to Smhb1. Testing against the wider range of isolates from this site was done, as it has previously been demonstrated that some phages are capable of infecting across several bacteria phyla [51,52]. None of the isolates tested were found to be susceptible to Smhb1, including *Salinivibrio* DV.

To establish the phylogeny of the *Salinivibrio* isolates, to determine the features of the prophage in BNH, and possibly to explain why DV was resistant to Smhb1, the genomes of both *Salinivibrio* isolates were sequenced (Table S1). Analyzing the genomes using GTDB-Tk showed that they were two separate species (*Salinivibrio kushneri* BNH and *Salinivibrio proteolyticus* DV) belonging to phylogroup I and III, respectively, as defined by de la Haba [53]. Given the specificity of most phages for their cognate host, the inability of Smhb1 to infect DV might be expected; however, further analysis indicated another plausible reason for the inability of Smhb1 to infect the isolate DV (see Section 3.5). No other prophages were identified in the genomes of *S. kushneri* BNH and *S. proteolyticus* DV using PHASTER.

### 3.2. Basic Genome Characterization, Phylogeny and Prophage Integration

The genome of phage Smhb1 is 32,826 bp, within the average length for *Peduovirus* genomes (33776 bp ± 3800 bp), and has a GC content of 50.8%, close to that of its host (50.5%). Forty-nine putative open reading frames were predicted (Table S2), and no function could be assigned to twenty-three of these following BLASTp comparisons to genes of characterized phages. No introns or tRNAs were identified on the genome of phage Smhb1. 

Tetranucleotide usage deviation analysis showed a Pearson correlation coefficient (PCC) of 0.77 between Smhb1 and the *S. kushneri* BNH genome and 0.75 between Smhb1 and *S. proteolyticus* DV. When using the TCS feature in JSpeciesWS, the highest correlation was to *Salinivibrio sharmensis* DSM 18,182 (PCC-0.776). Interestingly, many of the best hits were to *Yersinia* species; however, apart from phage L413C, *Peduovirinae* related phages that infect *Yersinia* sp. have only recently been deposited on the NCBI database (MT374855, MT374858, and MT374857). A search for prophages in some of these *Yersinia* genomes showed that each contained either a part of or a full *Peduovirus* prophage and, in some cases, multiple complete *Peduovirinae* phage genomes.

The phage was classified as part of the ICTVs efforts to name and classify the many new phage genomes discovered through next generation sequencing efforts. Smhb1 is currently the only member and, therefore, type species of the genus *Playavirus*, named for the geographic feature from which its host was isolated (https://tinyurl.com/saze2zth (accessed on 8 August 2021); 2020.117B.R.*Peduovirus*es).

Physical characterization of the Smhb1 genomic DNA consisted of digestion with restriction endonucleases (Figure S1) to establish the presence or absence of DNA modifications as a mechanism to avoid susceptibility to endonucleases encoded by BNH [54,55]. Such modifications usually involve the addition of a methyl or glycosyl group (Krüger & Bickle, 1983; Adams & Burdon, 1985). EcoRI (five sites), HindIII (five sites), HhaI (336 sites), and RsaI (61 sites) are insensitive to dam or dcm methylation and were able to digest the phage DNA. 

MboI (71 sites) is sensitive to dam methylation, and the phage DNA remained uncut. While the related phages K139 and PV94 encode their own N6 adenine methylases [56], no equivalent enzyme could be identified on the Smhb1 genome. *S. kushneri* BNH does, however, encode a DNA (cytosine-5) methyltransferase (dcm) as well as a DNA adenine methylase (dam), which may explain why MboI digestion was blocked.

We further investigated the relation of Smhb1 to other *Peduovirinae* using VIRIDIC, which showed that, of the viruses on the NCBI virus database, Smhb1 was the most similar to the recently sequenced *Vibrio alginolyticus* prophage Valm-yong1 (MN56393) at the nucleotide level and sharing 26% identity over the length of their genomes. A prophage found in *Salinivibrio siamensis* KP-1, however, appears to be its closest relative at 56.3% identity. 

**Figure 3:**
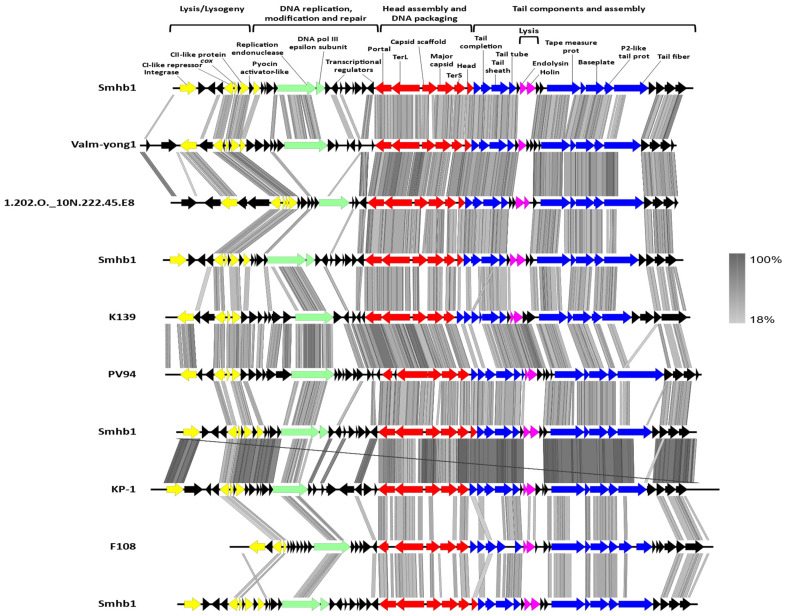
Genome organization and tBLASTx comparison of Smhb1 with its closest relatives.

ViPTree analysis and BLASTp was performed and showed that the majority of annotated proteins were similar to those of phages ValM-yong 1 (*Vibrio alginolyticus*), VD1 (*Vibrio diazotrophicus*), PV94 (*Vibrio vulnificus*), K139 (*Vibrio cholerae*), Kappa (*Vibrio cholerae*), 1.202.O._10N.222.45.E8, and VPUSM 8 (*Vibrio cholerae*) [57] (Figure S2). This was supported by the BLASTx analysis and alignment of these related genomes (Figure 3). 

None of the Smhb1 encoded proteins shared any amino acid similarity to proteins encoded by the podovirus CW02, one of the three published *Salinivibrio*-infecting phages, which belongs to the T7-like supergroup [24]. The other two *Salinivibrio*-infecting phages that have been described are the myoviruses UTAK [23] and G3 [58]; however, the genomes are unavailable, and no comparisons can be made. UTAK is currently propagated on *Vibrio hispanica* in culture collections, while G3 displayed a remarkably wide host range [58,59] demonstrating that phages that are known to infect *Salinivibrio* species can exhibit a wide host range.

Smhb1 inserted into the *S. kushneri* BNH *dusA* gene (tRNA dihydrouridine synthase) involved in the post translational reduction of uridine, which serves as an integration site for prophages and genomic islands in over 200 bacteria [60,61]. The integrase encoded by Smhb1, therefore, belongs to the family of *dusA*-associated integrases. The only other *Peduovirinae* virus that encodes a similar integrase is *Pseudoalteromonas* phage C5a (73% identity), which adds to the repertoire of integrases encoded by the *Peduovirinae* [62]. 

**Figure 4:**
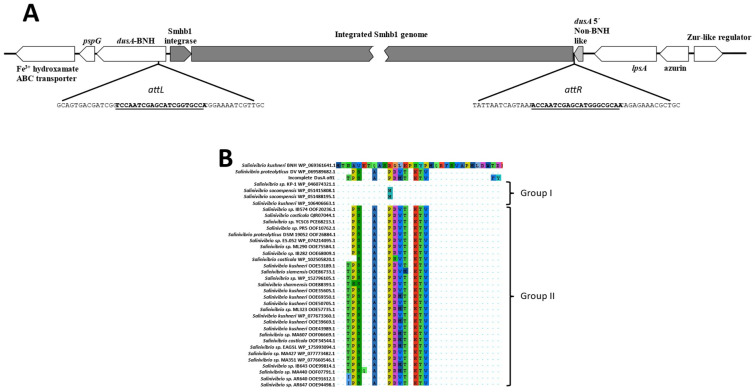
(**A**) Graphical representation of the integration site of the phage Smhb1 within the genome of the host, BNH. (**B**) Alignment of the *N*-terminal 32 amino acids of selected *Salinivibrio* DusA proteins showing the differences in the amino acid composition. Differences from the reference sequence (BNH top) are highlighted.

Using the consensus attachment sequences reported for this family of integrases [61], we identified the *attL* and *attR* sites for Smhb1 consisting of imperfect direct repeats flanking the inserted phage with the *attL* sequence located inside the *dusA* gene encoded by *S. kushneri* BNH, while the second repeat constitutes the last 21 bp of a 30 amino acid fragment of the N-terminal of a non- BNH *dusA* gene (Figure 4A). Analysis of the integrated genome showed another conserved 12 bp direct repeat (CAGTCTCCCTAT) with one repeat 26 bps upstream of the integrase gene and the second downstream of the non-BNH 5′-dusA gene region mentioned above (Figure 4A). 

These repeats lie roughly 180 bp apart on the phage genome as extracted from intact head particles and may have an undescribed role in phage integration. Investigation of the *attR* region showed that a small region between the crossover from the bacterial genome sequence and the start of Smhb1 genome appeared to harbor a 30 amino acid region of a *dusA* gene from a *Salinivibrio* strain other than DV or BNH. The arrangement of *dusA* fragments as depicted in Figure 4A is highly reminiscent of the arrangement seen by Farrugia and coworkers in *Acinetobacter baumannii* on the insertion of a genomic island [61]. 

The alignment of several complete *dusA* genes from *Salinivibrio* species indicated that the N-terminus can be grouped into two groups and that *S. proteolyticus* DV and *S. kushneri* BNH each fall into a different group with the *dusA* represented by the 30 amino acid stretch being in the second group (Figure 4B). This may be evidence to support that this phage has, in the past, infected a *Salinivibrio* species/strain other than *S. proteolyticus* DV or *S. kushneri* BNH (see CRISPR section below).

Immediately downstream of the *dusA* gene, we find the phage shock protein G (PspG) followed by a Fe^3+^-hydroxamate ABC substrate-binding protein. PspG is part of the phage shock response, a generalized membrane-related stress response system that is upregulated during phage infection [63,64], and the transporter is responsible for the uptake of hydroxamate siderophores. Iron uptake is one of the features identified as strongly downregulated when *pspG* is overexpressed [65]. 

The baseplate assembly proteins of P2-like phages incorporates iron ions, which served as examples when Bonnain, Breitbart, and Buck proposed their “Ferrojan hypothesis”, which was expanded on recently by Muratore and Weitz [66,67]. The predicted Smhb1 tail spike protein (gp49) does contain one H-T-H motif predicted to be the iron binding motif. In the Muratore and Weitz model, they considered the influence of iron availability on the ability of virus infection to change the host’s iron uptake strategies, and they concluded that viral infection and nutrient dynamics jointly shape the fate of microbial populations. 

However, this was seen from a purely lytic perspective and more complex interactions might be observed as, in the case of Smhb1, where lysogenic conversion may affect iron uptake. *S. kushneri* BNH does not have genes associated with siderophore production but does encode a siderophore uptake protein and would, therefore, act as a “defector” host in the model [67]. The location of the *pspG* gene and siderophore uptake transporter relative to the integrated Smhb1 phage suggests that phage integration may lead to modulation of iron uptake through this ancillary aspect of the phage shock response and siderophore uptake protein.

### 3.3. Lysis/Lysogeny

Holin and endolysin-like ORFs were identified on the Smhb1 genome together with a lysogeny module. Nilsson and co-workers investigated the arrangement and evolution of P2-like phage lysogeny modules and found distinct P2-like and phage 186-like lysogeny modules [62]. The arrangement of genes within the lysogeny module of most P2 phages is thought to follow a similar pattern *attL*-integrase-*C*-*cox*/*apl*; however, Smhb1 shares a lysogeny module of the 186-type with the genes arranged as *attL*-integrase-*cI*-*cox*/*apl*-*cII* (Figure 3). 

Apart from allowing the phage to escape host defenses, methylation, discussed earlier, may also be important for regulating the switch between lysis and lysogeny. Dalia and coworkers demonstrated that there are two undermethylated sites at the *cI* operator site located between the *cI* and *cox* genes in phage K139 and that binding of the *cI* repressor is likely responsible for undermethylation of these sites [68]. They further speculated that methylation of the site may not allow binding of *cI*, thereby, leading to lytic induction. We identified two canonical dam methylation sites in the region covering the *cI* gene and the intergenic space between *cI* and *cox* with one located 4 bp upstream of the translational start of *cI* and the other located 74 bp downstream of the translational start.

When compared with the NCBInr database, the Smhb1 endolysin, which contains a M15A peptidase motif, had higher similarity to endolysins from prophages in *Photobacterium*, *Vibrio*, *Serpentinomonas*, and *Pseudomonas* (>50% identity) and showed little homology to the proteins from K139, KP-1, PV94, and the *Pasturella*-infecting phage F108 with the closest *Peduovirus* encoded endolysin being that of *Pseudoalteromonas* phage C5a (32% identity). This likely demonstrates adaptation to a different cell wall structure of *S. kushneri* BNH. 

The holin belongs to class I with three transmembrane domains and no dual start motif. Phage holins are known to display greater sequence diversity than endolysins [69], and the holin from Smhb1 is no exception. BLASTp comparison of the Smhb1 holin with homologs on the NCBI viral database showed that it was only found in Smhb1 with more distantly related homologs (<43% amino acid identity) in *Vibrio* phage vB_VchM_VP-3213, *Vibrio* phage LP.1, *Oceanospirillum* phage vB_OliS_GJ44, and *Pseudomonas* phage 14-1 among others. 

Thus, the Smhb1 endolysin and holin are not conserved even among its closest relatives. One evolutionary requirement for holins is that they need to create a pore large enough to allow access of the cognate endolysin to the peptidoglycan layer in the cell wall [70]. It could be that the unique endolysin and holin of Smhb1 are co-evolved for effective lysis in *S. kushneri* BNH.

### 3.4. DNA Replication and Packaging

The model *Peduovirinae* phage, P2, replicates via a rolling circle mechanism following circularization of the genome through cohesive ends [71]. Replication is initiated by a phage encoded endonuclease (gpA) introducing a single stranded nick at the origin of replication (located within the gpA encoding ORF), which becomes covalently bound to the 5′ end of the nick site. The homologous protein in Smhb1 is encoded by gp15. Should replication in Smhb1 proceed as described for P2, we can expect the replication origin to be located within gp15. 

Evidence that the origin of replication is located here is the presence of the conserved nick site (*ori*) sequence in gp15, which appears to be conserved among the *Peduovirinae* (Figure S3). Cumulative GC-skew analysis has been used to predict the origin and terminus of replication for several DNA species [72,73] and, in Smhb1, showed a local minimum (origin) at 18,101 bp and a maximum (terminus) at 2901 bp (Figure S4). 

When assessing the cumulative GC-skew for the P2 phage, the tool predicts a local minimum at 29,401 bp, close to the experimentally determined origin (29,892 bp), suggesting an accurate prediction. Whether or not the local minimum in cumulative GC-skew identified for Smhb1 carries significance with respect to the role it might play in replication is yet to be determined.

The large and small subunit terminase genes are encoded by gp27 and gp30 and are responsible for the initiation of the packaging of viral DNA. The terminase large subunit is not only useful as a phylogenetic marker [74,75] but also as an indicator of the type of packaging mechanism employed by the phage [76,77]. This can be phage specific but generally follows one of a handful of models, and phages with similar terminase genes have been shown to package DNA in the same manner [76,77,78]. 

**Figure 5:**
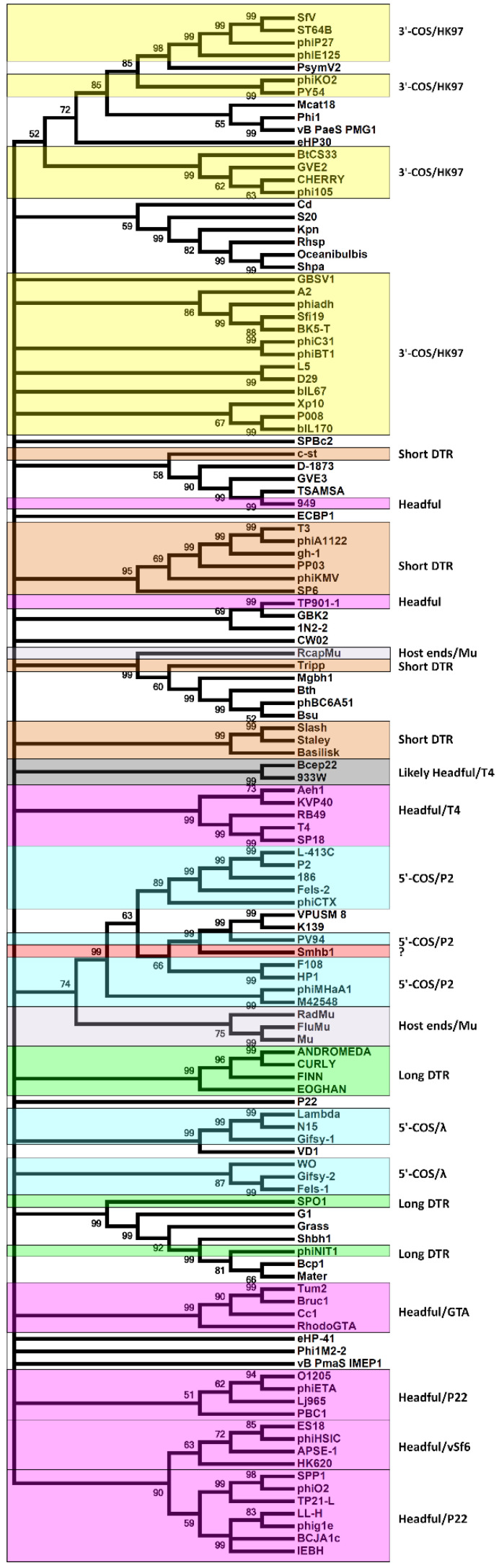
Phylogenetic tree comparing Smhb1 terminase large subunit to phage terminases for which the packaging mechanism is known. The evolutionary history was inferred using the Maximum Likelihood method based on the JTT matrix-based model. There was a total of 160 positions in the final dataset. The accession numbers for the top phage terminase hits are listed in supplementary data. The red star denotes the position of Smhb1 large terminase subunit in the tree.

In P2, the phage genome replicates as a single copy through a rolling circle mechanism instead of replicating as concatenated genomes as with phages, such as lambda, and these single genomes serve as the substrate for packaging [79]. Comparison of the Smhb1 terminase to others suggests that the DNA packaging mechanism phage Smhb1 employs is similar to that of P2, 186 and related phages, being cos-dependent (Figure 5) [57,80].

### 3.5. Late Gene Expression and Virion Protein Features

In phage P2, the expression of late genes is controlled through autoregulation by the Zinc-finger containing protein Ogr (an ORF located between the integrase and late control D family gene) of its own promoter whereas, in phage 186, this role falls to cI [81,82,83]. In phage K139, the ogr homolog is located between the portal protein and replication endonuclease, which is also the case for Smhb1 (Figure 3) [56]. Once produced, Ogr acts as an inducer of late gene expression, and the location of the Ogr homolog in K139 and Smhb1 may suggest a different timing of late gene expression compared with the enterobacterial phages P2 and 186.

The genomic organization of the head and tail assembly modules of phage Smhb1 show a close relationship to phage K139 with 11 characterized genes showing significant homology (Table S2). The capsid of P2-like viruses is encoded by at least four genes [80]. In Smhb1, these are encoded by gp26 (P2 gpQ portal protein), gp28 (P2 gpO homolog), gp29 (P2 gpN homolog), and the gp30 (P2 gpL homolog). A BLASTp analysis of the gp29 protein identifies the Phage_cap_P2 domain conserved among *Peduovirinae*, including K139, HP1, and HP2, which have been shown to be highly conserved in similarity [56]. 

Kapfhammer describes the capsid of P2-like phages as a DNA container that requires little or no adaptation to different hosts, whereas the tail formation genes show greater divergence [56]. This is ignoring the fact that these viruses find themselves in a range of physicochemical environments and that all proteins making up the physical structure of the virus would be under selective pressure under these conditions. 

A signature of halophilic protein adaptation is an increase in the use of Asp, Glu, Val, and Thr residues and a decrease in Lys, Ile, Met, Leu, and Cys as well as a reduction in the predicted pI value [84]. Analysis of the aligned concatenated moderate halophilic capsid, portal, head scaffolding, terminase large and small subunits, and sheath proteins compared against a non-redundant list of non-halophile *Peduovirinae* homologs did not show the same preference in amino acid usage or pI (Figures S5 and S6). 

The moderately halophilic phage proteins appeared enriched for His, Trp, Lys, Met, and Asn. This could be due to *Salinivibrio* and *Vibrio* species not being extreme halophiles such as those analyzed by Paul et al. [84]. We did note substantially increased Ala and Arg content of *Burkholderia* infecting *Peduovirinae* phages. No other discernable patterns related to amino acid composition were identified (enriched for hydrophobic residues, solvation potential, etc.).

### 3.6. Smhb1 Resistance in S. proteolyticus DV

As mentioned earlier, the phage Smhb1 was unable to infect *S. proteolyticus* DV. One possible defense mechanism is the presence of CRISPR spacer regions targeted against the phage [85,86]. In microbial hosts, CRISPR loci usually contain a combination of CRISPR-associated (*cas*) genes and non-coding RNA elements, which are responsible for the target specificity of CRISPR-mediated nucleic acid cleavage [87,88]. Seven CRISPR regions were identified in *S. proteolyticus* DV and three regions in *S. kushneri* BNH (Table S4), including the largest CRISPR array found among the *Vibrionaceae* to date at 179 spacers [89]. 

BLASTn analysis of all the spacers identified in both bacteria against the phage Smhb1 genome showed that seven spacers from *S. proteolyticus* DV were perfect matches to the genome. The spacers target several genes involved in phage replication, packaging, and virion morphogenesis (tail tube and capsid), which should ensure that no functional progeny can be reproduced in vivo (Table S5). 

As taxonomic classification of *S. kushneri* BNH and *S. proteolyticus* DV indicated that these are two distinct species, and this presents two scenarios for the presence of spacers targeting Smhb1 in *S. proteolyticus* DV [53]. According to McDonald et al. [89], many CRISPR arrays found in the *Vibrionaceae* are acquired through horizontal transfer and are found inside or on mobile elements (transposons, plasmids, and genomic islands). Thus, either the spacer regions targeting Smhb1 were inherited with the CRISPR array or Smhb1 can infect more than one species of *Salinivibrio*.

### 3.7. Accessory Genes

The K139 encoded G protein-like ORF, or Glo protein, is a secreted virulence factor of *Vibrio cholerae* [90]. gp4 on Smhb1 showed low but detectable homology (20% identity, 44% similarity) to the Glo protein from phages K139, VcP032, and VD1. The KP-1 prophage, PV94, and phage F108 have no such homologs. Although this could suggest that *Salinivibrio* species may act as reservoirs for phages harboring these virulence factors, the absence of a canonical CAAX motif at the C-terminal as was observed for the protein from φO18P. Apart from its role in virulence, the Glo protein was also shown to play a role in phage exclusion in K139, and gp4 may perform a similar function in Smhb1 [91].

## 4. Conclusions

We described phage Smhb1, a novel member of the family *Peduovirinae*. Despite being isolated from a unique habitat and having a unique nucleotide sequence, the conservation of amino acid sequences and P2-like modular layout of its genome demonstrates that the selective pressure to maintain P2-like features must be strong and present in the Eisfeld playa. The host range appears to be limited to *Salinivibrio* species; however, taken together, the data presented suggests that Smhb1-type phages could display a wide host range. Smhb1 serves to extend the adaptability window of the P2 phage model.

## Data Availability

The datasets presented in this study can be found in online repositories. The genomes have been submitted to NCBI and can be found under the GenBank accession numbers MEBP00000000, MEBQ00000000 and NC_047775.

## Supplementary Materials

The following are available online at https://www.mdpi.com/article/10.3390/microorganisms9102043/s1. Figure S1: Agarose gel electrophoresis of digested phage DNA. Lane one phage Lambda DNA cut with PstI used as a molecular marker, Figure S2: ViPTree analysis of Smhb1 encoded proteins compared with those of other *Peduovirinae* phages present on the Virus-Host DB: RefSeq release 93. The branch that Smhb1 (NC_047775.1) is on is highlighted in red, Figure S3: Alignment of origin of replication (ori) sequences from Smhb1 and related *Peduovirus*es. Only differences from the reference (Smhb1) are shown, Figure S4: GC skew analysis of the genome of phage Smhb1 and P2 to predict the putative replication origin (ori) and termination sites (ter) calculated using a window size of 1000 bp and a step size of 100 bp, Figure S5: Amino acid composition profiles comparing moderately halophilic *Peduovirinae* proteins to non-halophilic *Peduovirinae* encoded proteins ordered by observed difference, Figure S6: Predicted protein isoelectric point (pI) of selected *Peduovirinae* encoded proteins ranked from highest to lowest. Smhb1 predicted protein pI indicated in red and those of Vibrio infecting phages in green, Table S1: Summary of the sequence analysis report for the assembly of phage Smhb1, Table S2: CRISPRs and number of spacers identified in the genomes of Salinivibrio species DV and BNH using CRISPRFinder, Table S3: A summary of the BLASTx analysis of the ORFs of phage Smhb1. The + or – sign before the ORF number indicates on which strand the gene is found, Table S4: Number and sequence of CRISPR repeat regions identified in BNH and DV, Table S5: Sequence and genomic location of CRISPR spacers targeted against various ORFs on Smhb1 identified in DV.
