# Supplementary material for: Genomic Characterization of a Prophage, Smhb1, That Infects Salinivibrio kushneri BNH Isolated from a Namib Desert Saline Spring
Source: Microorganisms. 2021 Sep 28;9(10):2043. doi: 10.3390/microorganisms9102043 (PMC8537503; doi:10.3390/microorganisms9102043)
Supplement: Supplementary file 1 [file microorganisms-09-02043-s001.zip › microorganisms-1353552-supplementary.pdf]

SUPPLEMENTARY DATA

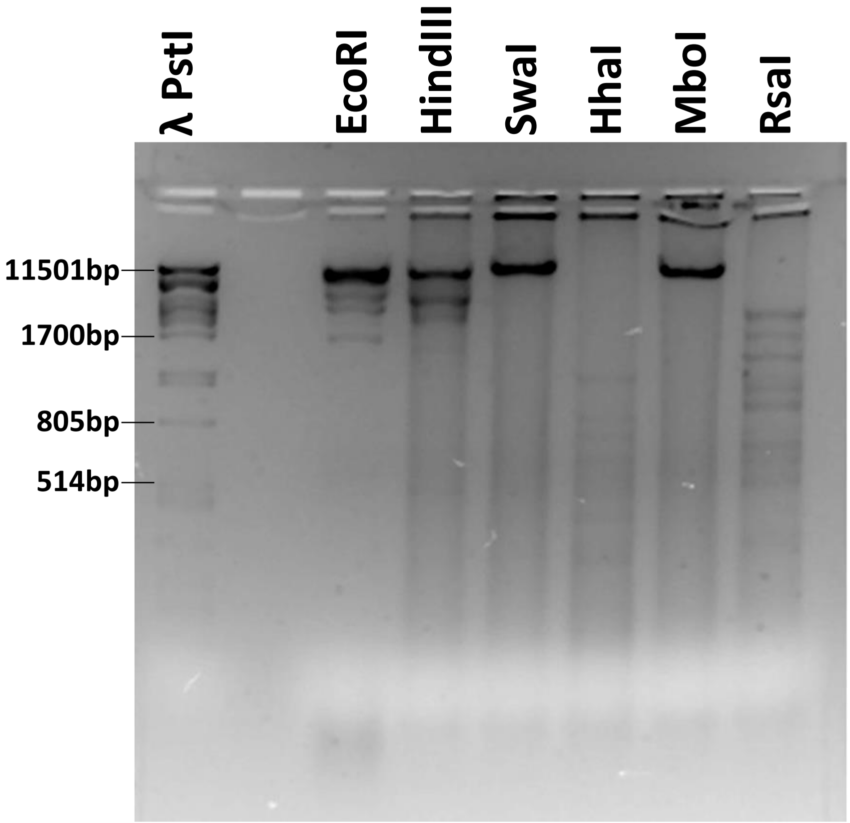

Figure S1. Agarose gel electrophoresis of digested phage DNA. Lane one phage Lambda DNA cut with PstI used as a molecular marker.

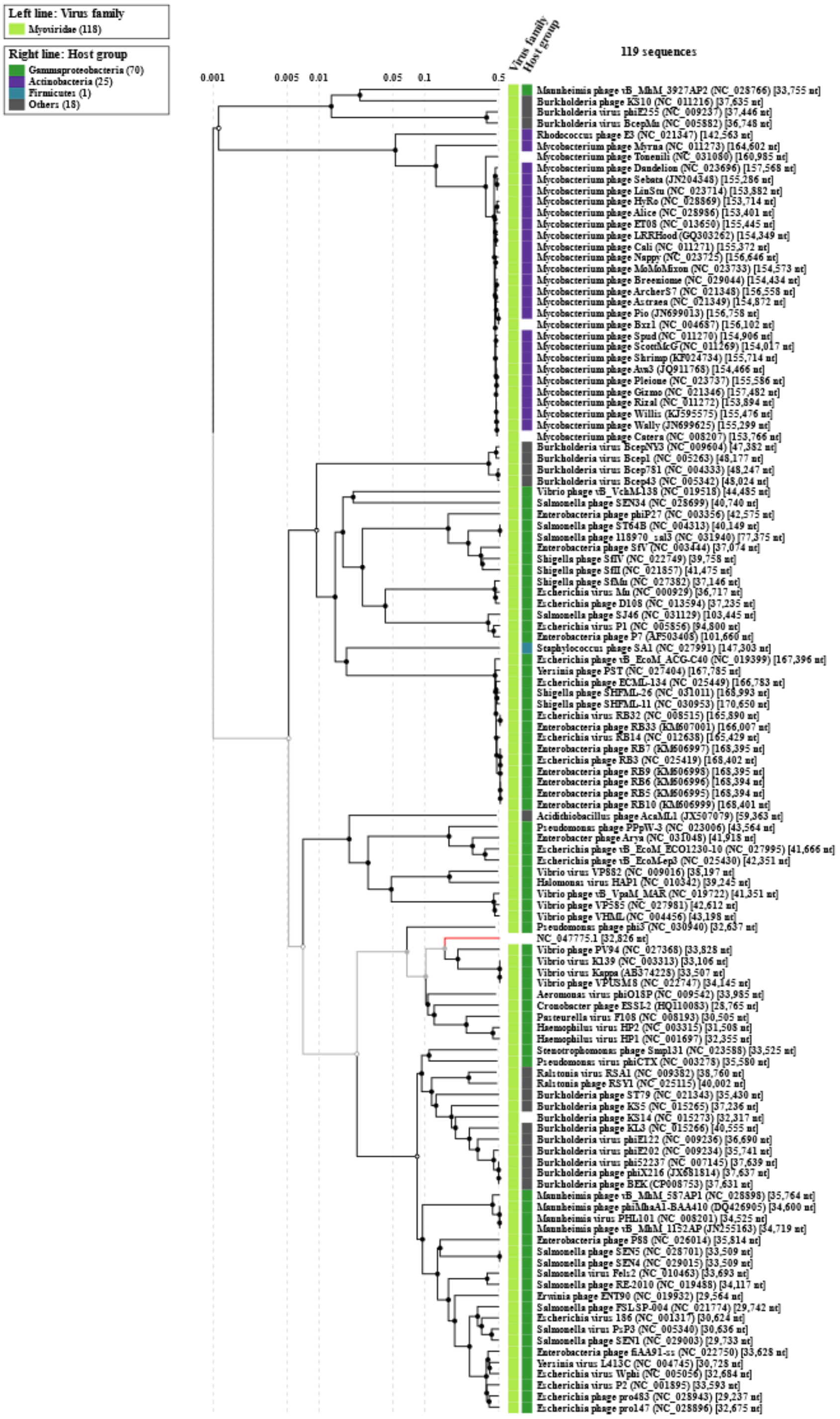

Figure S2. ViPTree analysis of Smhb1 encoded proteins compared with those of other *Peduovirinae* phages present on the Virus-Host DB: RefSeq release 93. The branch that Smhb1 (NC_047775.1) is on, is highlighted in red.

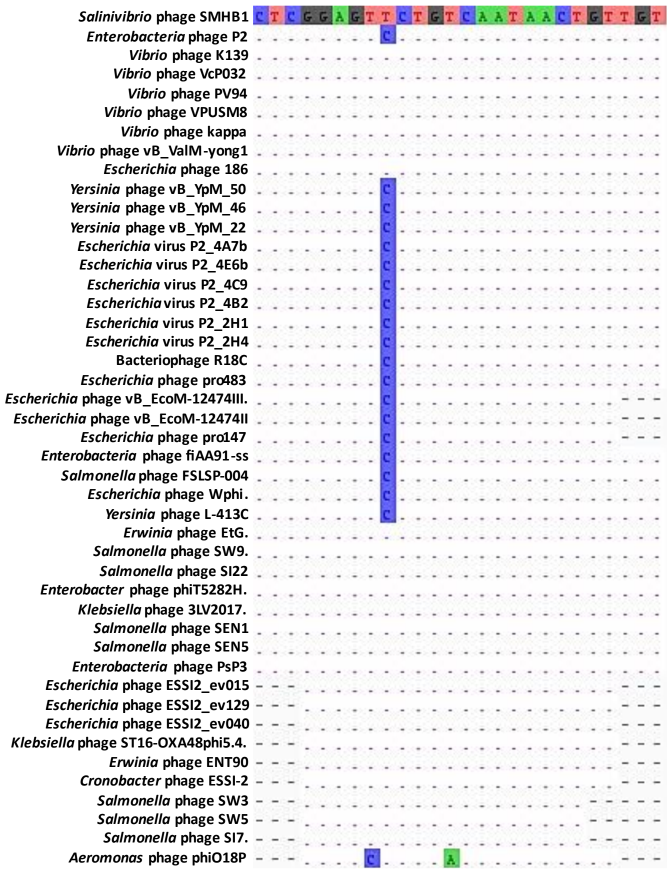

Figure S3. Alignment of origin of replication (*ori*) sequences from Smhb1 and related *Peduoviruses*. Only differences from the reference (Smhb1) are shown.

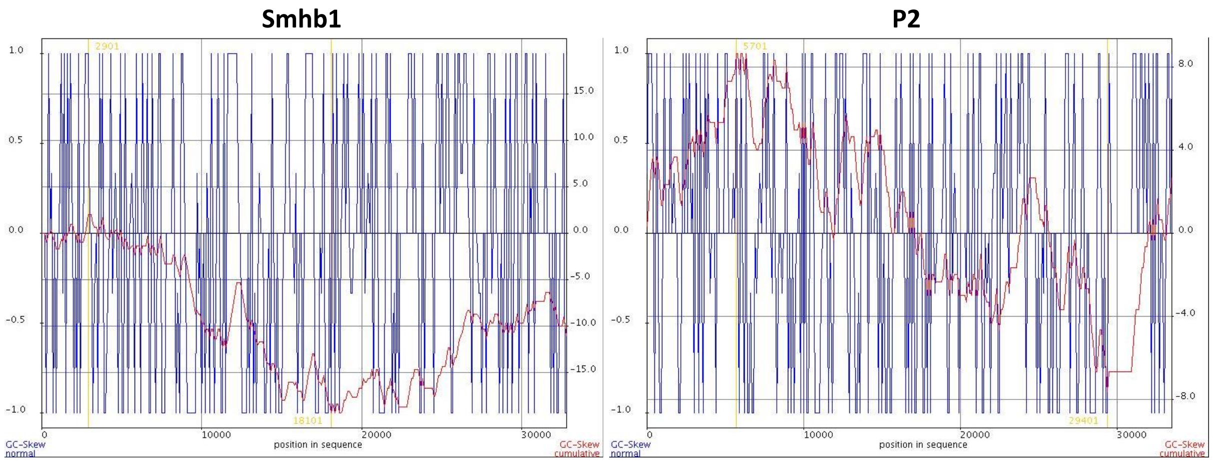

Figure S4. GC skew analysis of the genome of phage Smhb1 and P2 to predict the putative replication origin (ori) and termination sites (ter) calculated using a window size of 1000 bp and a step size of 100 bp

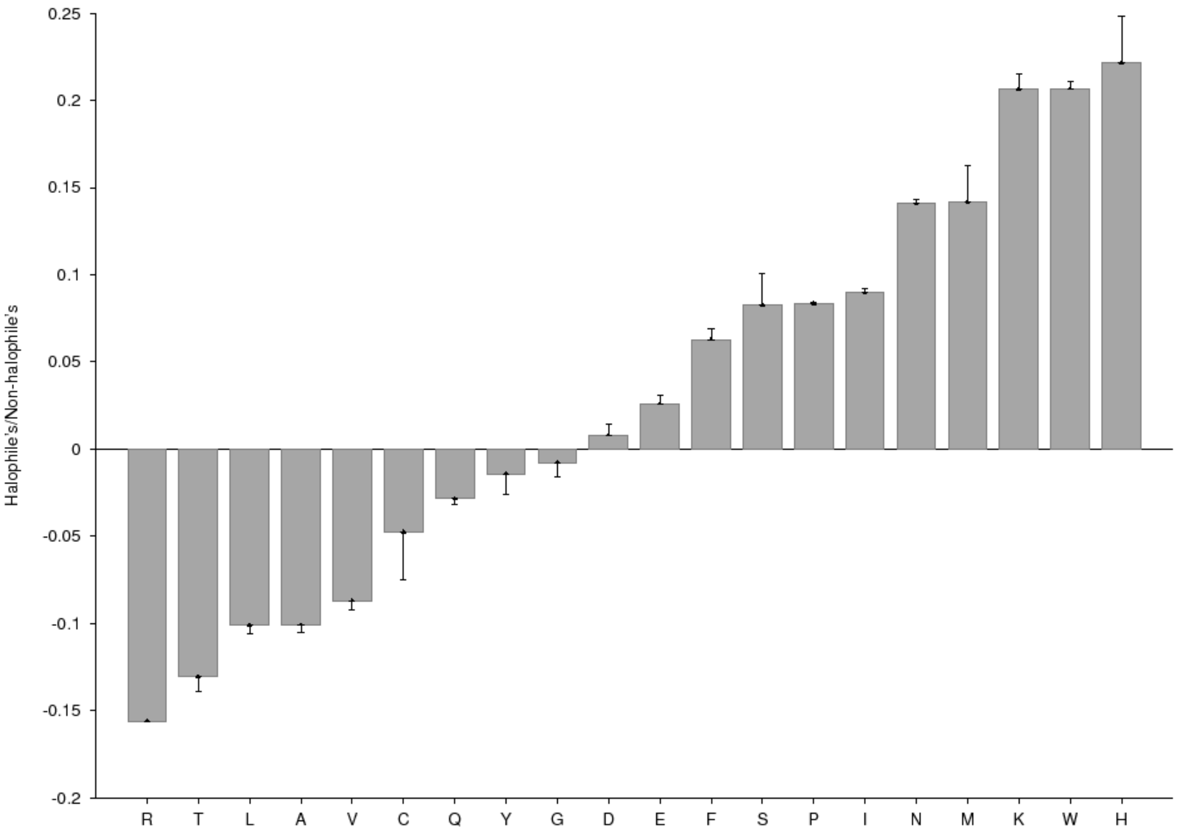

Figure S5. Amino acid composition profiles comparing moderately halophilic *Peduovirinae* proteins to non-halophilic *Peduovirinae* encoded proteins ordered by observed difference

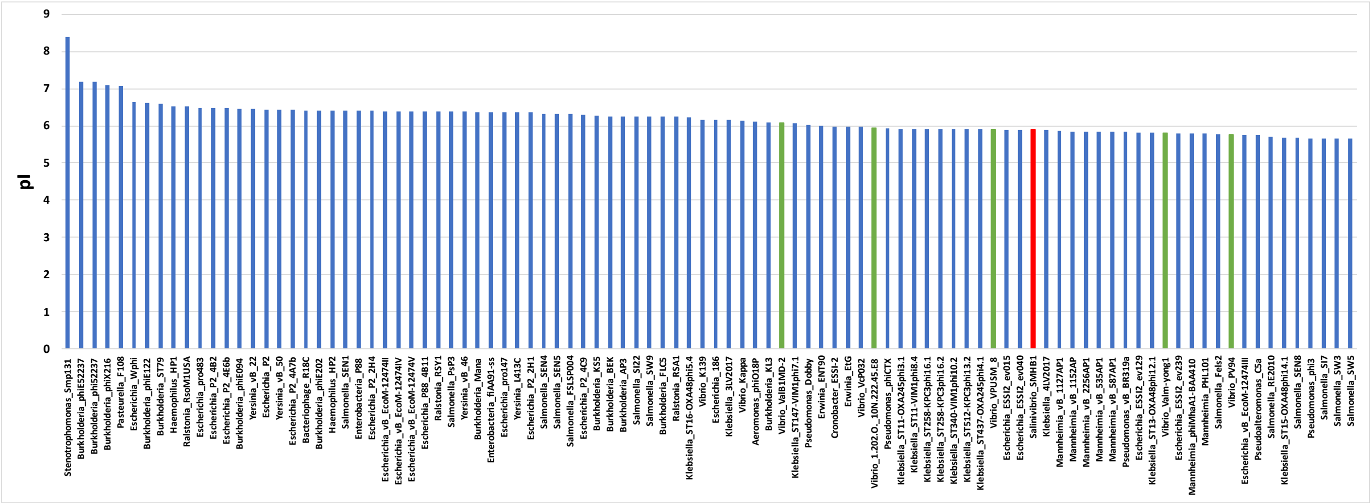

Figure S6. Predicted protein isoelectric point (pI) of selected *Peduovirinae* encoded proteins ranked from highest to lowest. Smhb1 predicted protein pI indicated in red and those of *Vibrio* infecting phages in green

Table S1: Sequencing analysis report for the assembly of phage Smhb1 and the genomes of DV and BNH

Attribute	*S. proteolyticus* DV	*S. kushneri* BNH	Smhb1
Total number of reads	1,681,207	2,029,522	543,053
Avg. Read length (bp)	273	279	288

Genome size (bp)	3,710,861	3,482,133	32826
DNA G+C (%)	49.8	50.5	50.8
Number of contigs >500bp	145	45	-
Largest contig (bp)	184,643	399,209	-
N50 (bp)	51,625	187,815	-
Avg. coverage	124x	211x	1991x
Number of proteins	3242	3051	49
Accession number	MEBP00000000	MEBQ00000000	NC_047775

Table S2:

ORF number / direction	ORF start – end position (bp)	Accession Number	Closest phage BLASTp hit(s)	Identities	Positives	E-value
gp01 / +	450-1514	YP_009786943.1	Integrase *Pseudoalteromonas* phage C5a	204/279 (73%)	244/279 (87%)	2e-157
gp02 / +	1617-2105	YP_009786944.1	Hypothetical protein	-	-	-
gp03 / -	2154-2663	YP_009786945.1	Hypothetical protein Lv-1_gp24 *Lactobacillus* phage Lv-1	31/88 (35%)	48/88 (54%)	0.001
gp04 / -	2715-3167	YP_009786946.1	Hypothetical protein	-	-	-
gp05 / -	3213-3812	YP_009786947.1	CI repressor *Vibrio* phage 1.202.O._10N.222.45.E8	83/199 (42%)	121/199 (60%)	9e-47
gp06 / +	3905-4105	YP_009786948.1	Regulatory protein *Vibrio* phage Valm-yong1<br>Cox *Pasteurella* virus F108	45/65 (69%)<br>21/53 (40%)	55/65 (84%)<br>34/53 (64%)	9e-28<br>2e-10
gp07 / +	4108-4266	YP_009786949.1	Hypothetical protein	-	-	-
gp08 / +	4294-4815	YP_009786950.1	Regulatory protein CII *Vibrio* phage 1.202.O._10N.222.45.E8	85/173 (49%)	118/173 (68%)	2e-53
gp09 / +	4829-5092	YP_009786951.1	Pyocin activator PrtN family protein *Pseudoalteromonas* phage C5a	49/84 (58%)	65/84 (77%)	6e-33
gp10 / +	5102-5506	YP_009786952.1	Protein of unknown function DUF5347 *Vibrio* phage Valm-yong1	37/99 (37%)	49/99 (49%)	2e-12
gp11 / +	5571-5780	YP_009786953.1	Hypothetical protein	-	-	-
gp12 / +	5790-5906	YP_009786954.1	Hypothetical protein	-	-	-
gp13 / +	5911-6372	YP_009786955.1	Hypothetical protein	-	-	-
gp14 / +	6381-6617	YP_009786956.1	Hypothetical protein K139p11 *Vibrio* virus K139	36/72 (50%)	51/72 (70%)	3e-20
gp15 / +	6614-9046	YP_009786957.1	Replication endonuclease *Vibrio* phage PV94	390/772(51%)	520/772(67%)	0.0
gp16 / +	9056-9610	YP_009786958.1	Putative exonuclease *Vibrio* phage vB_VpaM_MAR	147/182 (81%)	170/182 (93%)	1e-109
gp17 / +	9621-9965	YP_009786959.1	Hypothetical protein	-	-	-
gp18 / -	9998-10333	YP_009786960.1	Putative transposon-related DNA-binding protein *Vibrio* phage Valm-yong1<br>Helix-turn-helix transcriptional regulator *Vibrio* phage PV94	30/93 (32%)<br>39/103 (38%)	55/93 (59%)<br>59/103 (57%)	4e-12<br>3e-11
gp19 / +	10314-10439	YP_009786961.1	Hypothetical protein	-	-	-
gp20 / +	10563-10724	YP_009786962.1	Hypothetical protein	-	-	-
gp21 / +	10880-11275	YP_009786963.1	Hypothetical protein	-	-	-
gp22 / +	11340-11474	YP_009786964.1	Hypothetical protein Her_0050 *Vibrio* phage Her	23/34 (68%)	28/34 (82%)	2e-10
gp23 / +	11492-11941	YP_009786965.1	Hypothetical protein	-	-	-
gp24 / +	11938-12252	YP_009786966.1	Hypothetical protein	-	-	-
gp25 / -	12234-12671	YP_009786967.1	ogr/Delta-like zinc finger family protein *Vibrio* phage 1.202.O._10N.222.45.E8	77/150 (51%)	103/150 (68%)	8e-47

gp26 / -	12730-13788	YP_009786968.1	Portal protein *Vibrio* phage Valm-yong1	248/346 (72%)	288/346 (83%)	0.0
gp27 / -	13770-15563	YP_009786969.1	Terminase *Vibrio* phage ValB1MD-2	465/596 (78%)	514/596 (86%)	0.0
gp28 / +	15745-16704	YP_009786970.1	Capsid scaffolding protein *Vibrio* phage VD1	153/323 (47%)	188/323 (58%)	2e-80
gp29 / +	16704-17756	YP_009786971.1	Major capsid protein *Vibrio* phage VD1	209/346 (60%)	259/346 (74%)	2e-151
gp30 / +	17759-18475	YP_009786972.1	Terminase small subunit *Vibrio* phage Valm-yong1	138/239 (58%)	187/239 (78%)	1e-100
gp31 / +	18574-18981	YP_009786973.1	Head completion/stabilization protein *Vibrio* phage PV94	77/139 (55%)	93/139 (66%)	2e-43
gp32 / +	18981-19457	YP_009786974.1	Tail completion protein R *Vibrio* phage Valm-yong1	93/155 (60%)	121/155 (78%)	4e-66
gp33 / +	19441-20097	YP_009786975.1	Virion morphogenesis protein *Vibrio* phage 1.202.O._10N.222.45.E8	152/219 (69%)	178/219 (81%)	3e-103
gp34 / +	20100-21221	YP_009786976.1	DUF2586 family protein *Vibrio* phage PV94<br>Tail fibers protein *Vibrio* phage Valm-yong1	264/373(71%)<br>264/376(70%)	308/373(82%)<br>305/376(81%)	0.0<br>0.0
gp35 / +	21221-21676	YP_009786977.1	Tail tube protein *Vibrio* phage Valm-yong1	111/151 (74%)	130/151 (86%)	2e-77
gp36 / +	21695-21901	YP_009786978.1	Hypothetical protein	-	-	-
gp37 / +	21906-22319	YP_009786979.1	Peptidase M15A uncultured Mediterranean phage uvMED	75/128 (59%)	96/128 (75%)	2e-50
gp38 / +	22322-22906	YP_009786980.1	Hypothetical protein VP3213_05 *Vibrio* phage vB_VchM_VP-3213	78/183 (43%)	112/183 (61%)	6e-35
gp39 / +	22907-23155	YP_009786981.1	Hypothetical protein HYQ07_gp37 *Vibrio* phage Valm-yong1	32/75 (43%)	56/75 (74%)	7e-16
gp40 / +	23152-23418	YP_009786982.1	Tail assembly chaperone *Vibrio* phage PV94	43/85 (51%)	60/85 (70%)	9e-25
gp41 / +	23607-25733	YP_009786983.1	Tail tape measure protein *Vibrio* phage PV94	423/707 (60%)	518/707 (73%)	0.0
gp42 / +	25730-26059	YP_009786984.1	DUF2590 family protein *Vibrio* phage PV94	74/107 (69%)	94/107 (87%)	1e-49
gp43 / +	26052-27239	YP_009786985.1	Baseplate J/gp47 family protein *Vibrio* phage PV94	248/392 (63%)	311/392 (79%)	0.0
gp44 / +	27226-27840	YP_009786986.1	Hemolysin *Vibrio* phage PV94	89/162 (55%)	119/162 (73%)	3e-61
gp45 / +	27837-30023	YP_009786987.1	ORF35 *Vibrio phage* O395<br>Putative tail fiber protein *Vibrio* phage VPUSM 8	175/273 (64%)<br>175/273 (64%)	213/273 (78%)<br>213/273 (78%)	2e-119<br>4e-117
gp46 / +	30023-30481	YP_009786988.1	Hypothetical protein ICP12011A_066 *Vibrio* phage ICP1_2011_A	45/81 (56%)	60/81 (74%)	5e-24
gp47 / +	30485-31030	YP_009786989.1	Tail-collar fiber protein *Vibrio* phage Valm-yong1	107/181 (59%)	135/181 (74%)	6e-73
gp48 / +	31027-31713	YP_009786990.1	Hypothetical protein HOU21_gp40 *Vibrio* phage 1.202.O._10N.222.45.E8	131/226 (58%)	172/226 (76%)	9e-103
gp49 / +	31704-32435	YP_009786991.1	Tail spike *Pseudoalteromonas* phage C5a	152/254 (60%)	182/254 (71%)	9e-102

Table S3:

**Bacterium (Strain)**
Salinivibrio costicola (LMG11651)
Vibrio casei (LMG25240)
Vibrio splendidus (LMG19031)
Vibrio chagasii (LMG21353)
Vibrio atlanticus (LMG24300)
Vibrio aestuarianus (LMG7909)
Vibrio harveyi (LMG4044)
Vibrio neocistes (NRRL B-1037)

Table S4:

**DV**		**BNH**	
**CRISPR repeat consensus**	**Number of Spacers**	**CRISPR repeat consensus**	**Number of Spacers**
GAAGCGAAAGCTAAGCGTGAAGC (23 bp)	5	TTTCTAAGCTGCCTGTGCGGCAGTGAAC (28 bp)	36
TGTTCACTGCCGCACAGGCAGCTTAGAAA (29bp)	3	TTTCTAAGCTGCCTGTGCGGCAGTGAAC (28 bp)	41
GTTCACTGCCGCACAGGCAGCTTAGAAA (28bp)	9	GTGTTCCCCGTGCCCACGGGGATGAACCG (29 bp)	47
GTTCACTGCCGCATAGGCAGCTTAGAAA (28bp)	35		
TTTCTAAGCTGCCTGTGCGGCAGTGAAC (28 bp)	24		
CGGTTCATCCCCGTGGGCACGGGGAACAC (29 bp)	42		
GTCGCGCCTCCCGCAGGCGCGTGGATTGAAAC (32bp)	178		

Table S5:

**Spacer**	**Smhb1 genome coordinates**	**Smhb1 gene recognized**
TTAGCTTCTGCGGGGTGGTTCGCGCAGCGGTAGC	22081-22114	Hypothetical protein
ACATCAACGGCGTGCCCTATCTGCGTGAAGACGAG	21621-21655	Putative tail tube protein
ATTGTTGCCTGCTGATCAGGTCAAAGGGCAAGTTA	16871-16905	Capsid protein
CGGTGTATATGCCATATGCGGCAATAGTACGCC	15540-15572	Terminase large subunit
GATGGTGTCGTTGCCGACACCGATTTTTTGTC	8883-8914	Replication protein
AGCGGCCCCACGTATTCGCGTGTTTTGGTTTT	8000-8031	Replication protein
GAATGCGGCGTTGTAGTTCACCACCCATTCGA	6922-6953	Replication protein
